# Supplementary material for: Priority-based transformations of stimulus representation in visual working memory
Source: PLoS Comput Biol. 2022 Jun 2;18(6):e1009062. doi: 10.1371/journal.pcbi.1009062 (PMC9197029; doi:10.1371/journal.pcbi.1009062)
Supplement: S1 Text — Fig A. Example 7-hidden-unit RNN trained with input following the basis function used to build IEMs in Wan et al. [1]. Shown is the 2D visualization of the LSTM hidden layer activity of this RNN. The network architecture and training procedure are identical to the 7D RNNs reported in the main text with the exception that the inputs are not one-hot vectors; instead, they are specified by the IEM basis function: R = sin6(x) (e.g., for stimulus #3, input vector is [0.0156, 0.4219, 1, 0.4219, 0.0156, 0]). Note that these results are qualitatively similar to RNNs reported in the main text (Fig 4). Fig B. Generating circular input for 60-hidden-unit RNNs. Each point on the circle can be characterized by an angle relative to the easternmost point of the circle. The coordinates of these points within the 2D space on which this circle lives are given by [cos θ, sin θ]. To construct input vectors used in our RNN model, we mapped each stimulus orientation θ to the corresponding point on the circle at 2 * θ. The multiplication by 2 is necessary to match the periodicity of the input vectors to the periodicity of the oriented grating stimuli, which have a period of 180° (i.e., the stimulus at θ is equivalent to the stimulus at θ + 180°). Fig C. Empirical test for presence of stimulus information in WM. (A) Time course of stimulus averages projected into the PMI subspace from an example 60D RNN. Data points are colored based on item n’s identity. We used the 3 timesteps prior to the presentation of n to construct a baseline distribution of dispersion values using a bootstrapping procedure. Visually, one can see stimulus information collapsing in the PMI subspace across the three timesteps that follow timestep n + 2, colored squares added to identify them for panel B. (B) The baseline distribution of dispersion values, with red dashed line indicating the 95th percentile criterion. Magenta, green and orange lines indicate the dispersion values from timesteps delay 3:1, delay 3:2, an [file pcbi.1009062.s001.docx]

**Supporting Information**

**
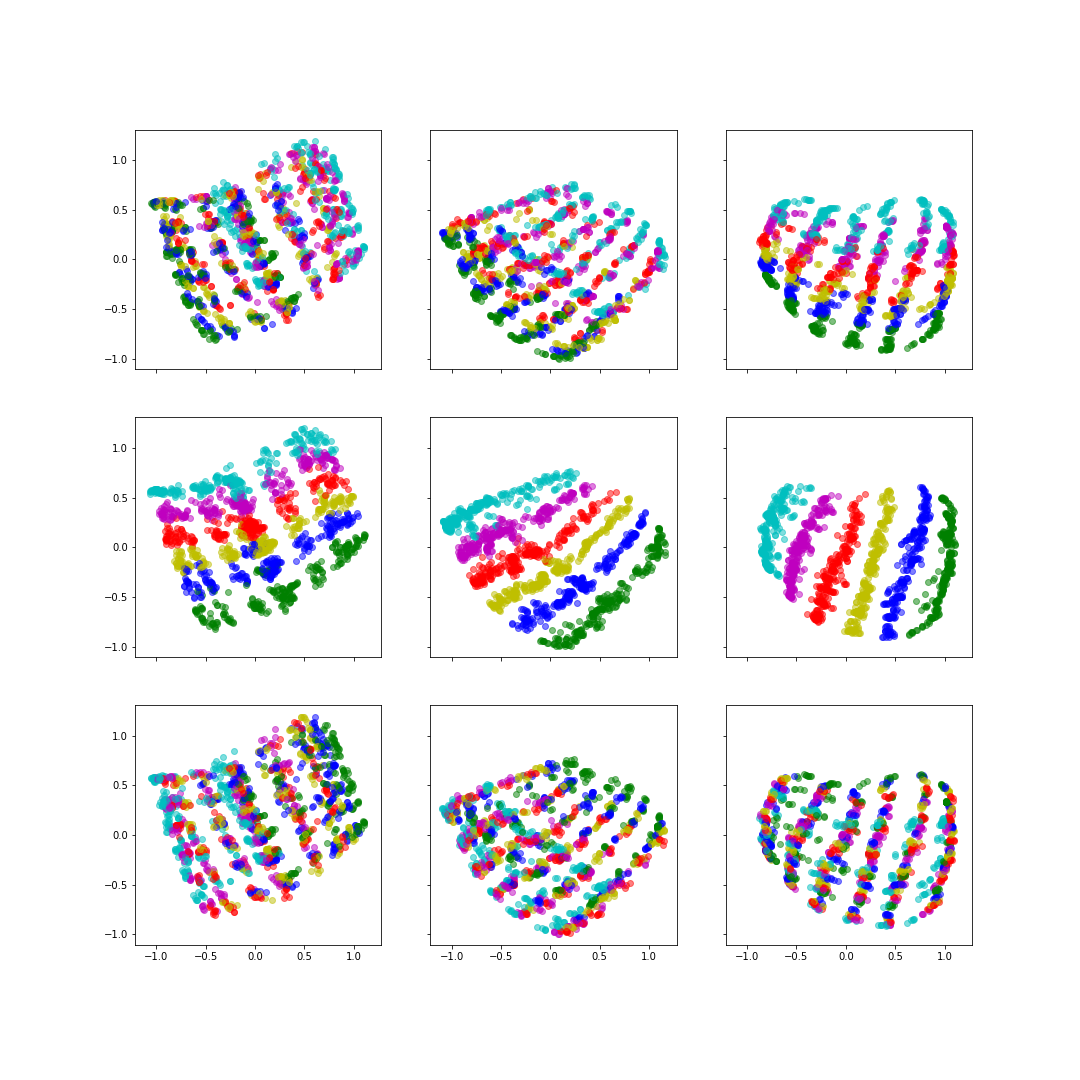
**

**Fig A.** Example 7-hidden-unit RNN trained with input following the basis function used to build IEMs in Wan et al. [1]. Shown is the 2D visualization of the LSTM hidden layer activity of this RNN. The network architecture and training procedure are identical to the 7D RNNs reported in the main text with the exception that the inputs are not one-hot vectors; instead, they are specified by the IEM basis function: $R=\sin^{6} (x)$ (e.g., for stimulus #3, input vector is [0.0156, 0.4219, 1, 0.4219, 0.0156, 0]). Note that these results are qualitatively similar to RNNs reported in the main text (Fig 4).


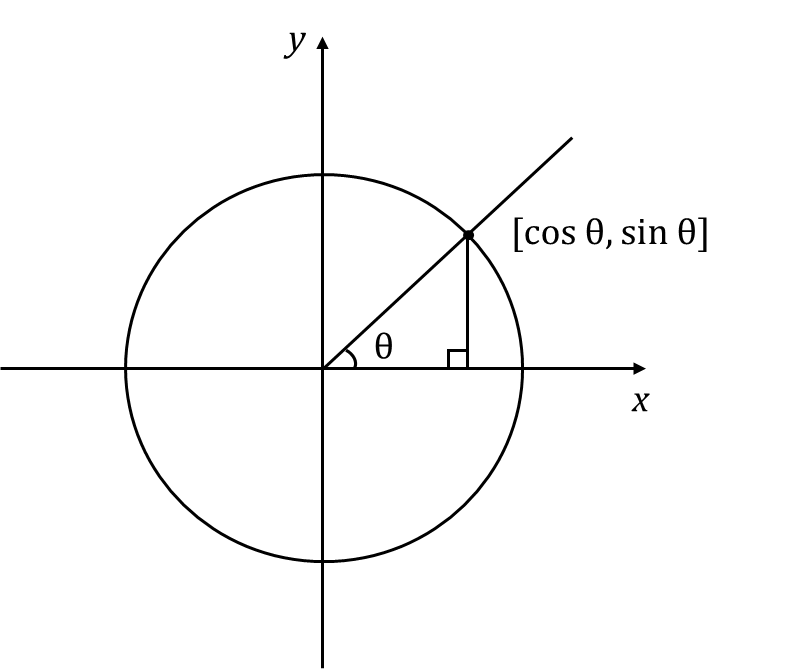


**Fig B.** Generating circular input for 60-hidden-unit RNNs. Each point on the circle can be characterized by an angle relative to the easternmost point of the circle. The coordinates of these points within the 2D space on which this circle lives are given by [cos θ, sin θ]. To construct input vectors used in our RNN model, we mapped each stimulus orientation θ to the corresponding point on the circle at 2 * θ. The multiplication by 2 is necessary to match the periodicity of the input vectors to the periodicity of the oriented grating stimuli, which have a period of 180° (i.e., the stimulus at θ is equivalent to the stimulus at θ + 180°).

**
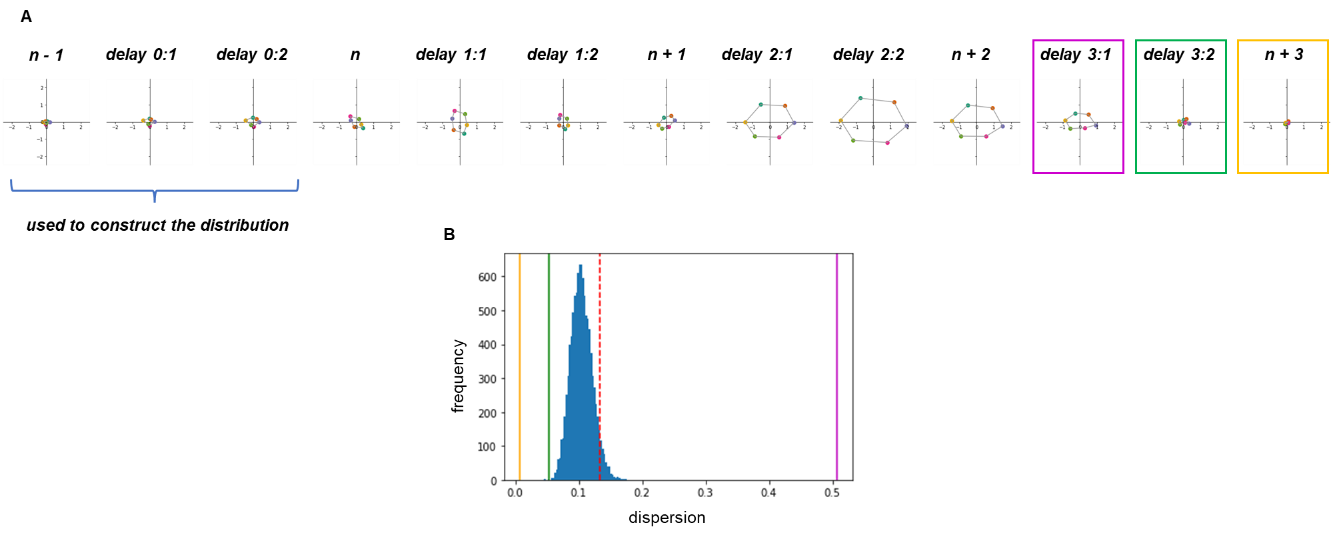
**

**Fig C.** Empirical test for presence of stimulus information in WM. (A) Time course of stimulus averages projected into the PMI subspace from an example 60D RNN. Data points are colored based on item *n*’s identity. We used the 3 timesteps prior to the presentation of *n* to construct a baseline distribution of dispersion values using a bootstrapping procedure. Visually, one can see stimulus information collapsing in the PMI subspace across the three timesteps that follow timestep *n + 2*, colored squares added to identify them for panel B. (B) The baseline distribution of dispersion values, with red dashed line indicating the 95^th^ percentile criterion. Magenta, green and orange lines indicate the dispersion values from timesteps *delay 3:1, delay 3:2*, and *n + 3,* respectively.


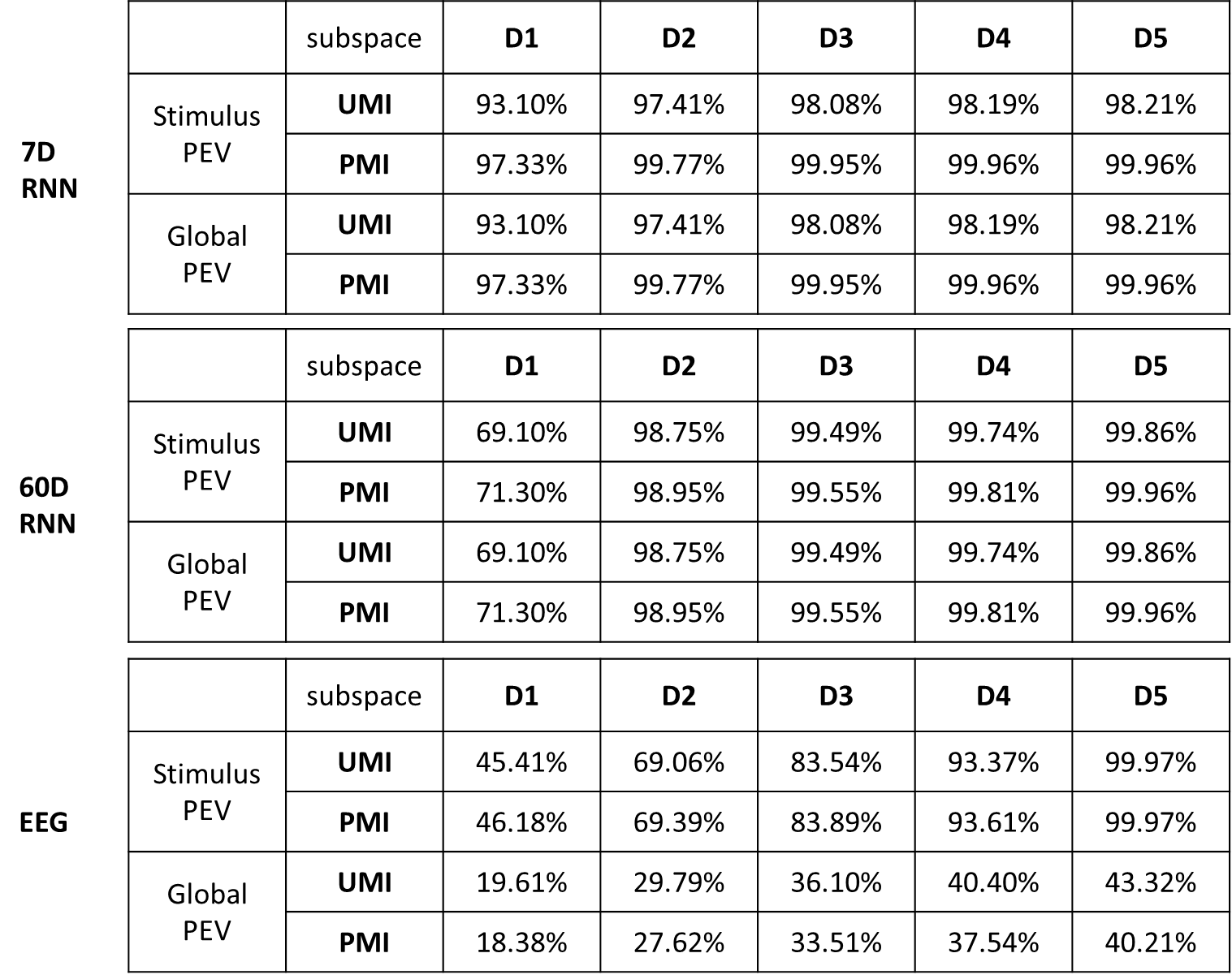


**Table A.** Cumulative percent variance explained (PEV) by top dPCs of the UMI and PMI subspaces for 7D RNN, 60D RNN and EEG data. The percentages of both stimulus and global variance explained are shown.

References

1. Wan Q, Cai Y, Samaha J, Postle BR. Tracking stimulus representation across a 2-back visual working memory task. R Soc Open Sci. 2020 Aug;7(8):190228.
