# Supplementary material for: Priority-based transformations of stimulus representation in visual working memory
Source: PLoS Comput Biol. 2022 Jun 2;18(6):e1009062. doi: 10.1371/journal.pcbi.1009062 (PMC9197029; doi:10.1371/journal.pcbi.1009062)
Supplement: Attachment — Submitted filename: WanMenendezPostle_2022_response_to_reviewers.pdf [file pcbi.1009062.s002.pdf]

Responses to review comments

In this file we have pasted-in the reviews of PCOMPBIOL-D-21-00800 in this Times New Roman font, and interleaved our replies *in this italicized sans serif font*.

Reviewer #1: In this manuscript, Wan and colleagues address a timely question that could be of interest to the readership of Plos Computational Biology. However, I find the initial framing of the paper confusing (and long) and I think the analyses seem preliminary. Below I provide a few suggestions that the authors might consider to improve the paper.

Framing

There have been intense debates about the neural basis of prioritized vs unprioritized working memory maintenance, with diverse evidence pointing to storage in different brain areas, reversed neural codes (the hypothesis addressed here) or 'activity-silent' mechanisms.

It is important to stress that some of these hypotheses are mutually exclusive, in contrast to what the authors claim.

We disagree with this statement, but please see our response to the next paragraph of the review.

For example, if a memory is stored in an *active* reverse code, does it make sense to talk about a *silent* code? If it is reversed, it is not silent, since silent is the opposite of active. Of course, there are many biological processes co-occurring in the brain that could be considered "silent" (i.e. not based on spiking activity), such as forms of plasticity, channel adaptation, etc. An active reverse code could then coexist - and most likely it does - with a silent process. However, the likely possibility that an active code might coexist with silent mechanisms is not evidence for WM maintenance through silent mechanisms. This confusion is pervasive in the field and this manuscript somehow helps perpetuate it. The work described here is a good proof of concept that a reverse code does not need any sort of "silent" mechanism and thus the paper could have been framed as such. Indeed, without touching the results section, one could frame the paper as disqualifying the storage of UMI in activity-silent codes, similar to what has been done for reactivations seen in fMRI: "Restoration of fMRI Decodability Does Not Imply Latent Working Memory States" by Schneegans & Bays in Journal of Cognitive Neuroscience (2017) 29 (12): 1977–1994

*Although there are some specific statements here, pertaining to scientific questions, that we might quibble with, the point that's most important for us to address in this context is: "*This confusion is pervasive in the field and this manuscript somehow helps perpetuate it." *We disagree strenuously with this characterization, for the simple reason that our paper is very clearly NOT about putative activity-silent mechanisms of WM maintenance, nor about the possible co-existence of activity-silent with active mechanisms. Indeed, the construction "activity-silent" only appeared in 3 instances in our initial submission: twice in the first paragraph of the paper (to provide background context) and once in the References (because it's in the title of a paper that we cite). Thus, the suggested reframing of the paper cannot work, because the data that we present simply do not address the questions that the reviewer is emphasizing here.*

Nonetheless, because we understand that reviewers serve as a proxy for a journal's readership more broadly, we see that it is important that we establish more clearly what our paper IS versus what it IS NOT about, and so have rewritten the end of the first paragraph of the paper as follows:

"At the time, these results were interpreted by some as being consistent with the idea that whereas prioritized memory items (PMI) are held in an active state, UMIs may be maintained as "activity-silent" traces encoded in synaptic weights (Barak & Tsodyks, 2014; Stokes, 2015). Although this possibility remains a topic of vigorous debate (Schneegans & Bays, 2017, Sprague et al., 2016, Christophel et al., 2018, Stokes et al. 2020] the present report does not relate directly to this question. Instead, the focus here is on more recent empirical results suggesting that, rather than producing a decline to baseline, deprioritization may produce a representational transformation of an item into a different, but still active, format. (We will return to a consideration of activity-silent models in the Discussion.)"

Thus, we have moved the consideration of the fact that an activity-silent mechanism could, in principle, co-exist with one or more active mechanisms, to the Discussion.

In general, I think an explicit discussion of this apparent contradiction would be valuable to clarify the current confusion. For example, how come the UMI decoding in Rose et al was at chance levels, but here, a below-chance code is found empirically and modeled with RNNs without short-term plasticity? Isn't the proposed RNN model here in contradiction with Rose et al, whereas the authors say: "UMIs may be maintained as "activity-silent" traces encoded in synaptic weights"?

Instead, the authors gloss over the controversy and ignore papers that consistently show (including their own EEG work and now modeling) that UMI are stored in active codes.

The truth is that, at this time, we don't have a good answer for why earlier studies (including Rose et al., 2016) didn't find evidence for an active but transformed representation of the UMI. However, these comments have prompted us to think carefully about these questions, and helped us develop a new "meta interpretation" of our approach, which is that it provides a means of assessing data, and potentially helping to interpret the results of decoding/encoding analyses, that is data driven, quantifiable, and that is not dependent on whether decoding/encoding was even successful. Thus, for example, data sets like the Rose et al. (2016) data that have yielded no evidence for above chance-level decoding of the UMI are just as amenable to analysis with dPCA as are the data that are the focus of this study. Thus, the methods that we introduce here might be able, in future work, to help address some of the controversy and confusion that does exist in this literature.

Analyses

The authors show interesting rotations in the RRN model, both by quantifying it and visualizing it in a 2-D principal component space. They also quantify similar rotations in EEG data; however, without showing the respective visualization (why?).

The 2-D PCA visualization of the RNN results plots all trials, which can yield clear visualization of representational structure thanks to the small trial-to-trial variability in the RNN architecture.

The same is not true, however, for the EEG data, which contain large trial-to-trial fluctuations that render trial-wise visualization uninformative.

I found the explanations/interpretations in the captions and the text (just 2 pages for results!) unsatisfactory.

For example, Figure 4 A and B look completely different, while the purpose of this study is to highlight their similarities. Improving this figure would be key to send the main message across for quick readers.

Yes, other reviewers have also called attention to Figure 4 and made helpful suggestions for how to better illustrate the comparison between RNN and EEG. However, this figure relates to the rotation analysis and therefore has been removed from the revision.

Figure 2 is copied from the original EEG study, but never replicated in the RRN. Generating an equivalent figure for simulated data would also make the main point more convincing. On the other hand, Figure 3, which is very difficult to follow, is never replicated in the EEG data.

With regard to these figures, other reviewers also raised concerns and/or questions, and so we have changed both considerably. (See details in responses to other reviewers; additionally, please note that Figure 3 from initial submission is now Figure 4.)

In response to the substantive point about replicating IEM results in the RNN, we have changed some of the text from the Introduction to make explicit why this would not help us address our question:

"From the perspective of the framework of Marr and Poggio (1976), both of the models reviewed above were intended to address the phenomenon of opposite results between UMI and PMI at the implementational level (e.g., why does the IEM reconstruction flip?). Our interest in this report, however, is not to understand how different conditions might influence the behavior of MVPA or IEM. Rather, our interest is at the algorithmic level of analysis: Are shifts in priority status accompanied by systematic transformations of neural representation? Thus, although MVPA and IEM produced the results that gave rise to the priority-based remapping hypothesis, they are poorly suited to evaluating it, because they don't permit direct measures of neural representation (Gardner & Liu, 2019; Liu et al., 2018; Sprague et al., 2018, 2019).

It is not clear how the reverse code emerges in EEG (I guess training on AMI but testing on UMI?).

The redesigned Figure 2 now illustrates explicitly (and specifies in the figure legend) that the reversal is produced with a model trained on the delay period of the 1-item delayed-recognition task.

The paper is motivated by and centered around the finding of inverted decoding. Yet, it is not clear how rotations can lead to such inverted decoding.

We hope that our revisions now make it clear that although this paper is indeed motivated by findings of inverted decoding, it is not "centered around" them. For example, how or why the creation of a "UMI subspace" that is partly overlapping the "PMI subspace" (the new results, which have superceded rotation [see responses to Reviewer 2]) might lead to inverted decoding

is a question one would ask if fundamentally interested in "how does MVPA/IEM work?", but for us, it is the discovery of the subspaces in the RNN data, and then the confirmation that the EEG data undergo a priority-based transformation that is qualitatively similar to what is observed with RNNs, that are of primary interest.

I guess inverted decoding in EEG signals would mean that AMI are stored in some area(s) while the UMI are stored in other(s)?

This is a possibility that we can't rule in or out with scalp-level EEG. However, we did (and continue to) note in the Discussion that in the Libby and Buschman (2021) study the "sensory axis" and the "memory axis" were represented by the same population of neurons in auditory cortex. Panichello and Buschman (2021) also explicitly call attention to this aspect of their results from PFC. Finally, we also note that, in studies with fMRI, priority-based remapping occured in the same posterior regions that also represent the PMI (van Loon et al, 2018; Yu et al., 2020).

At a minimum, it should be shown in what conditions the rotations and inverted encoding do not emerge. For example, would increasing the number of neurons (now 7!) still bring about these rotations?

A major new element in the revision is to replicate the 7-neuron results with RNNs with 60 LSTM units, so as to match the dimensionality of the EEG data. Additionally, the revised Methods also notes that RNNs with as many as 256 LSTM units showed performance very similar to the 7-unit networks that we continue to report in this revision.

Would one get the same rotations if the RNN were trained on an abstraction of the Rose task? If so, what does that tell us of the activity-silent interpretation of that paper? It is thus not very clear what was learned with this novel modeling work. The main purpose of training RNNs is to go beyond what is done in the data and propose mechanisms for an otherwise descriptive finding. The simple observation that similar dynamics occur both in the data and network is unsatisfactory to me and I guess as well for a general computational readership.

We are completely in accord with the reviewer about the main purpose of training RNNs. Although we had felt that we had achieved this, the initial submission evidently didn't convey it as effectively as needed. A combination of elements in these reviews – particularly R4's request for a more explicit 'walk-through' of an item's trajectory through a trial, and this reviewer's admonition (below) that we incorporate explicit consideration of the recent study from Panichello and Buschman (2021) – led us to articulate the new framing of our process that is now laid out in the final paragraph of the Introduction of the revised manuscript:

"Our approach was to train RNNs to perform the 2-back task, then first use Principal Component Analysis (PCA) of the activity of the RNN's hidden layer to visualize its representational dynamics. This revealed a smooth rotational transformation of stimulus representations over the course of the trial (Figure 4). This trajectory was consistent with what would be expected of a series of priority-based transformations of representational formats as stimuli transitioned functional roles from memory probe to UMI to PMI. However, PCA does not allow for the isolation and quantification of variation attributable to specific task dimensions (of particular interest here, priority status and the match/nonmatch decision). Therefore, we treated these observations as a hypothesis-generating step, and carried out three additional sets of analyses. First, we established the validity of these hypotheses in the RNN data by submitting them to demixed

Principal Component Analysis (dPCA; Kobak et al., 2016) -- a procedure that allowed for the identification of dimensionality-reduced subspaces specific to the probe, UMI, and PMI states of representation, as well as one specific to the decision – then quantifying the geometric relations of these subspaces and to one another as well as the temporal dynamics of the representational geometry within these subspaces. Second, we replicated these procedures with 60D RNN data. Finally, we treated the 60D RNN results as a priori hypotheses that we tested with the EEG data from Wan et al. (2020). The results of these hypothesis tests provided novel insights about priority-based transformations of stimulus information that are carried out by the human brain."

With regard to the Rose task, those experiments were carried out with decoding at the category level, which moves us even further away from the ability to study stimulus representation. So we don't think that returning to the specific details of that task would be a sensible thing to do. On the other hand, an important property that the Rose task shares with many others (including, e.g., Yu et al., 2020) is the unpredictability of the retrocue. Indeed, we did (and still do) identify the importance of extending the work presented here to a retrocuing task, in the Discussion.

General comments, as I re-read the paper

The introduction and discussion has excessive description of previous experiments, even defining variables that are never used throughout the paper:

"the basis function parameters for memory strength (φ), gain (γ), receptive field width (μ), and receptive field centers (δ) were varied. "

Point taken, we have removed these definitions of variables from another study. However, we do feel that (as is the case with the brief mention of the activity-silent hypothesis in the Introduction) it is important to contextualize the present work in relation to what has been done previously.

On the other hand, the results were too short. The authors could consider reducing the introduction and discussion and clarifying the results further.

The results have been expanded considerably, in part due to the requests for more details, in part to accommodate the results from analyses suggested by other reviewers, and in part by the addition of 60D RNN data.

Overall, I feel there are too many figures to show one simple - however interesting - point, which is that RNNs show rotations, as seen in EEG data. For example, figure 6 can be substituted by one short sentence. I had a hard time jumping from figure to figure while most figures could be collapsed into one figure.

This figure relates to the rotation analysis and therefore has been removed from the revision.

Why so few networks (and neurons!)? Figures would look much more convincing if many more networks would populate Figure 5, for example.

The network behavior was highly consistent across the 10 trained networks, and adding more wouldn't have changed the results in any appreciable way. Additionally, those results are now supplemented with the results from the 10 additional RNNs with 60 LSTM units.

The technical details about the RNN are not sufficiently clear. For example, the authors only used 7 neurons but an input of 6 dimensions. How much do the results depend on these choices? I would consider using bigger networks and including a diagram of the network architecture as well as the task used for training in Figure 1.

As mentioned above, RNNs with various numbers of hidden units (up to 256) generated similar results, and we have added this to the manuscript. We have also now included a schematic diagram of the network architecture as well as example stimulus and target output sequences used for training (Figure 3).

The authors discussed the results of Libby and Buschman (2021), here mice passively listen to auditory stimuli, to great extent. There is no mention of another work from the same lab that directly investigated the neural mechanisms of UMI vs AMI. This study seems extremely relevant to the modelling work produced here and I would recommend discussing it.

*Yes, the reviewer is referring to Panichello and Buschman (2021; doi.org/10.1038/s41586-021-03390-w) and we agree that these findings are also extremely relevant. Indeed, after re-reading it we also see how referring to it will help address points raised by other reviewers (e.g., R4: "*trying to translate [priority-based remapping] intuitively into what that means in terms of how the brain represents the stimuli.") *Please see our reply to this comment from R4 for specifics of how we now discuss the findings from Panichello and Buschman (2021) in the revised manuscript.*

Reviewer #2: Wan et al re-analyse EEG signals during a working memory 2-back task, and compare the EEG decoding with the hidden layer of a LSTM RNN trained to perform the task. This is a very hot topic and an interesting new approach. Notably they introduce a new metric, the "rotation index" which helps to quantify representational rotations in dimensionality-reduced data. The rotation may be of crucial importance in protecting information in WM from interference.

I enjoyed reading this manuscript and it has a great deal of novelty. I have mainly minor clarificatory points that I think might improve the paper.

1. The procrustes solution for a rotation assumes the mapping is a rotation rather than a scaling/negation. In other words, if the data showed a flip, this fitting would "force" it into a rotation. Note that a 180 degree rotation is the same thing as a "flip" of both the projected components (i.e. reflection on both axes), so it is possible that this kind of "flip" (scaling by negative value) might fit reasonably well. Importantly, a situation where there was *no* rotation but just a scaling, would be well-fitted by a rotation of zero. I may have misunderstood their permutation method, but it seems that a rotation of zero would give a significant rotation index?

For these reasons it would be nice if the authors compared the rotation R with a simple scaling (or even a combination of rotation*scaling). After all, this is the alternative hypothesis they flag up in the intro.

This is a very astute observation, and based on it we did try fitting scaling and scaling + rotation transformations between UMI and PMI means. And this approach did, indeed, yield better fits -- unlike rotation, scaling can reduce error from the different extent of dispersion between the UMI

and PMI data, and this new analysis shows that it is, in fact, differing degrees of scaling (and of dispersion) that account for the majority of the difference between them. Because scaling + rotation transformations did not appreciably improve the fit over scaling along, we are just reporting the results with this simpler model.

2. p5 para1 - "Notably, these “flipped” (or “opposite”, van Loon et al., 2018) states are not less active relative to the PMI representations, rather, they are different."

This sentence in brackets is unclear. In what way were they "different"? It seems the authors are suggsting why it was not simply a polarity change, but that doesn't seem with the IEM reading out a flipped response.

The reason for inclusion of this sentence has been obviated by a change that we’ve now made earlier in the Introduction (in response to R1) and so we have removed it.

3. p12 first para: "Namely, we added an extra stimulus event following the stimulus n trial. We only plotted..." This sentence was hard to follow: why was an extra event added, what was the stimulus? What is an 'overlapping' stimulus event and why are those trials not plotted? Maybe it can be illustrated on fig1.

Because trials in the 2-back task overlap (e.g., the [n n+1 n+2] trial overlaps with the [n-1 n n+1] trial), we had originally only plotted half the trials from the experiment (e.g., [n n+1 n+2], [n+2 n+3 n+4], [n+4 n+5 n+6], etc.) This is no longer the case, and now for this figure (previously Figure 3, now Figure 4) we plot all 3200 trials from the experiment. Therefore, the offending sentence no longer appears in the revised manuscript.

4. Might be helpful to adopt uniform naming of timpoints and reflect them in Fig1 e.g. I assume "delay 1:1-2" is the frame after "n" in Fig1?

The issue here is that from the perspective of task events, there is one unfilled gap separating each stimulus presentation (i.e., one “delay”). From the perspective of the RNN stimulations, however, there are two timesteps during each delay: “delay 1” spans timesteps delay 1:1 *and* delay 1:2. *We realize, however, that the specific detail about how the delays are parsed by the RNN is unnecessary (and unnecessarily complicating) for Figure 1, and so we’ve changed that. Additionally, we realize that trying to shorten references to “delay 1:1 and delay 1:2” to “delay 1:1-2” was unintentionally cryptic, and so we’ve removed all instances of the “1-2” shorthand from the revision.*

4. [continued] Fig 2 may need a bit more explanation, colormap legend, and meaning of "response" y-axes. Would a schematic, rather than this complex figure, be better?

In response to this (which is echoed by other reviewers) we’ve revised this figure extensively.

5. p16 line 1: "subspaces might be overlapping" - unclear what "overlapping" means for a pair of linear subspaces. Do the authors mean they may share one of their two axes? intersect?

Similarly, p21 second para, "confirming that these two subspaces overlap" - I am not sure of the logic why the presence of rotations in both directions confirms the spaces overlap. It may be that there are two orthogonal spaces, both of which have rotations? [Perhaps this can be determined from the inner products of the columns of the two W matrices.]

By "overlapping subspaces", we mean that PMI variability can be found in the UMI subspace and vice versa. Indeed, we have found the angle between UMI and PMI subspaces in the EEG data to be 60.70°, suggesting they are partially overlapping. We have now deleted the referred sentence to avoid confusion.

6. I really like the clever definition of the rotation index, which will be useful to future research. The authors could highligh this as part of the result/impact of this paper. Fig 4 could even visualise the rotated UMI against the PMI, or rotated PMI against the UMI, to show how the degree of alignment determines this index.

We appreciate this sentiment, a lot of mental sweat equity went into developing it. However, stated above, because scaling has superseded rotation as a better characterization of the transformational trajectory, we have opted, for this revision, to remove content related to the RI. However, at reviewer and editor discretion we'd be happy to, for example, mention it briefly and include the details as supplementary material.

7. There are three interesting ways the model deviates from the data. a) The data shows a strong 2-dimensional code (second PC is useful) for both the UMI and PMI. This doesn't seem to match the model. The authors should discuss why this might be. b) It is interesting that the PCA fails for EEG data but not for the RNN - could the authors discuss why this might be? c) are the EEG rotation angles significantly larger than the RNN's? I think these differences are of potential interest.

a) *We want to be cautious about ascribing functional significance to this difference, and now include the following in the Discussion: "It is important to note that the RNN modeling is not intended to simulate EEG data or the human brain, which has vastly different structural and functional architecture from this RNN. This may limit what can be interpreted from direct comparisons between the two sets of results. For example, because of the relative simplicity of the RNN architecture, and the absence of many sources of noise that are characteristic of EEG (e.g., physiological noise, uncontrolled mental activity, measurement noise), the variability and SNR of the two signals differ markedly. This may partly explain why, in our analyses, EEG variability is distributed across more dPC dimensions than RNN, which is largely one-dimensional (Figure 5 and 6, Supplementary Materials S2)*

b) *We suspect that it's due to the "cleaner" status of the RNN data, as indexed by the discrepant PEVs for the 1st dPCs of UMI and PMI in RNN vs. EEG data. Here's one just-so-story that seems plausible to us: The dPCA shows that whereas the majority of UMI contraction occurs during the first half of the trial, the majority of PMI expansion occurs during the second half of the trial. Perhaps the PCA, in effect, collapses across these two and extracts from them one smoothly rotating component? The same doesn't happen with the EEG data, because transitions in its 1st dPCs are not as prominent as they are in the RNN data?*

c) *This observation is obviated by the fact that rotation has been shown to not be the best way to characterize the data.*

7. Fig 3: do the axes show y_UMI or y_PMI?

I am guessing UMI because there is better separation for n+1. How was the "stimulus coding axis" determined? Panel S4A was helpful in understanding this and I wondered if it could be incorporated into fig4 somehow. I think figure 6 is redundant given fig 5?

In Figure 3 (now Figure 4), each data point is labeled according to stimulus n so it takes the status of UMI at delay 1:1 and delay 1:2 and of PMI at delay 2:1 and delay 2:2. The “stimulus coding axis” is purely schematic, i.e., from “eyeballing” the PCA visualization. The previous Figures 4-7 relate to the rotation analysis and therefore have been removed from the revision.

8. p24: dPCA "does not make assumptions about the representational structure of stimuli" -- it assumes the stimuli are represented linearly among neurons.

dPCA, as a dimensionality reduction technique, doesn’t assume how the stimuli are represented among neurons, whether linearly or nonlinearly, but does have the limitation that it finds directions of variability only in linear subspaces, as the reviewer alludes to.

The authors should be congratulated on a very interesting study.

Reviewer #3: Thanks for giving me the opportunity to contribute to the review process. This is a very interesting, novel and well written study on a candidate mechanism underlying the retention of unattended memory information (UMI). While there is a surge of evidence that the UMI reconstructions (i.e., colors, orientations etc.) are flipped relative to the attended (PMI) ones, it is not known how this is achieved at the mechanistic level. The authors provide a timely and important answer to this question. They model WM performance on a 2-back task using RNN-based LSTM models. The dynamic evolution of the hidden layers is inspected in detail using dimension reduction methods (PCA and dPCA). They convincingly show that a rotational remapping process characterizes the UMI-to-PMI state changes as index by RI. Interestingly, a similar rotational remapping was observed when dPCA was applied to EEG data (from a 2-back task). Overall, I believe this study is an important contribution to the field and has the potential of further inspiring new theoretical and methodological insights. Below I have listed some (mainly) clarification questions and (minor) comments that need to be addressed before publication.

The authors provide extensive details about PCA, dPCA and RI. Information about the RNN-LTSM is however sparse. Given its central role in the manuscript, the authors should provide more details about the model in the manuscript. While readers will have access to the materials on osf and where most of the questions below will find their answers, I could not consult the following information:

- Please define the RNN/LSTM more precisely, as it is currently difficult to understand how it is constructed. By definition, the application of LSTM implies there were forget units, remember units, sigmoids, and tanh functions in the hidden layer. If so, or otherwise, please provide this information in the methods.

- As far as I can interpret the model, there are multiple inputs, that are sequentially dependent, thanks to the recurrent nature of the network in the hidden layer. Given that the trial event is a concatenation of n, n+1 and n+2 along with the 2 delay periods each, it is not clear whether there is only 1 output (many-to-one), that of the n+2 target, or multiple outputs, that of each individual event: n, n+1 and n+2 along with the 2 delay periods (many-to-many). A graph depicting the model architecture could be helpful.

We agree that this is important information, and have added more detail about the RNN-LSTM. This includes:
- A newly added figure (Figure 3) that diagrams the RNN model architecture.
- We use default LSTM units in the Pytorch package (which has now been clarified in the text). Please refer to the PyTorch documentation for details. There is one output for each timestep (both stimulus presentation and delay). Target output for stimulus presentation timesteps is either 1 (match) or 0 (non-match) and that for the delay period is always 0 because the network does not output any decision. Please refer to Figure 3B for example input and target sequences for further clarification.

- Whether and how hyperparameters were optimized: any dropout or regularization?

Batch size and learning rate are arbitrarily decided: there is no basis for suspecting that varying these hyperparameters will affect the result. Varying the number of hidden units (up to 256) generated similar RNN behavior and representational patterns. No dropout or regularization was done.

- Whether the input states of a non-match and a delay period -that are both set to [0 0 0 0 0 0]- are functionally the same?

This reflects a confusion of input with output states, and we have made two changes that we believe will preempt this for readers of the revised manuscript. The first is the newly added Figure 3 It clarifies that <u>inputs</u> are always 6D, because there are 6 possible stimulus values, and for delay timesteps the input is set to [0,0,0,0,0,0] (except for the first two timesteps of each sequence, when no stimulus was presented). For stimulus presentation timesteps the input state was a one-hot vector with only the stimulus unit activated -- and 2/3 of these did not match the item presented two-back, and thus elicited an output corresponding to a non-match decision. Figure 3 also clarifies that <u>outputs</u>, generated by the single output unit, could take a value of [0] or [1]. (Thus, it doesn't make sense to say that the input state of a non-match is set to [0 0 0 0 0 0].) Second, the architecture of the RNN dictates that during training, cost is minimized only on timesteps with output generated (e.g., during a nonmatch response) thus in a sense only target output values on those timesteps have functional consequences. We included the following in the text: "The output unit took on a value at each timestep, with target output values of 0 during each delay timestep, of 0 for stimulus presentation timesteps presenting a non-matching stimulus, of 1 for stimulus presentation timesteps presenting a matching stimulus (i.e., a stimulus matching the item presented two items previously)."

- Ten RNNs were used (N=10). Please provide information on how these 10 RNNs vary. Do they vary in terms of their architecture, parameters, or train/test instances?

All RNNs trained had the same architecture, hyperparameters and training/test sequences. The only thing that differs across networks is the random initialization of the RNN weights. This information has now been added to the manuscript.

- Please explain why 2 delay periods were installed (does this reflect the time points before and after the mask in 2-back task? Or does this number relate to the length/duration of the interval?)

The 2 delay timesteps were installed to assess the stimulus representation in early vs late delay (we used the later delay timestep because we initially reasoned that representation in late delay

will be more stable and more reflective of the prioritization status given the comparison happens in the following time step). We also did simulations where the number of delay time steps were varied. The results were qualitatively the same although the more delay timesteps there are, the "slower" the rotation is.

The discussion provides plausible interpretations to the findings and draws connections to the literature (e.g., Libby & Buschman, 2021). Additionally, the limitations of the current study are addressed along with interesting future directions. I believe that the authors should also discuss potential reasons why rotational remapping was only revealed in the subspaces obtained from the dPCA alone and not with generic PCA. This is an empirical question that is probably beyond the scope of the current manuscript. My concern is that dPCA, which maximizes between-group (PMI vs UMI) variability and minimizes within-group variability, orthogonalizes the high dimensional feature space relative to the PMI/UMI conditions separately. Consequently, the subspaces that are optimally responsive to a specific condition may result in a flip or rotation in the other condition (potentially induced by the dPCA orthogonalization?).

dPCA finds dimensions that maximize variability between stimulus conditions and thus the observed rotation captures rotations in the stimulus representational space. However, PCA confuses many stimulus-irrelevant sources of variability which is not ideal to reveal the rotational structure in the stimulus-relevant subspaces. This explains why rotational remapping was only revealed in the dPCA subspaces but not with PCA. (Also see response to Reviewer 2, #7.)

We have conducted an alternative permutation test where we permute the stimulus labels before conducting dPCA. If rotational remapping is an artifact of the dPCA algorithm, we would expect to find rotations for all permuted cases and fewer subjects demonstrating significant rotations. However, this new permutation method generated the ***same*** *result. In addition, the fact there are subjects with no significant rotations (even with dPCA applied) argues against the possibility that the rotation is induced by the dPCA procedure.*

Finally, does rotational remapping predict any behavior? The correlation between RI and ACC/d' cannot be achieved due to ceiling performance and this is something that the authors have addressed in the discussion. However, I was wondering whether the authors have tried to look at any relation between RI and speed? The reason to establish such a link is to bridge the gap that exists now between the model and human data. The LSTM nicely models the 2-back performance, and the rotational remapping is derived from the hidden units' activity in PCA and dPCA subspaces. So, the model's inner workings are inherently coupled to performance. In analogy to this, any link between the RI and speed would be interesting to further support the functional role of rotational remapping.

Following the reviewer's interesting idea, we conducted an exploratory analysis by correlating RI and response time across all subjects, but failed to find a significant relationship when taking all subjects into account. We hope to address questions of brain-behavior relationships in future work!

Minor:

- It would be better to spell out LSTM (for the first time) for the sake of clarity for a broad readership. Same for ANN in the discussion.

LSTM ("long short-term memory") and ANN ("artificial neural network") now spelled out.

- The following sentence is related to the RNN, but the referenced figure is about the IEM data. Is this correct? (p13) 'As with the RNN data, after excluding the first two stimuli from each block there were 126 stimulus events and hence 125 trials per block (Figure 2).'

This is actually in reference to EEG data, and we have rephrased to say "As is the case with RNN" to make this clearer.

- RNN training and testing: "The activity timeseries of the LSTM hidden layer units from all 3200 trials (16 trials x 200 sequences) in the training data set were extracted for subsequent analyse" (P11) Is this correct? Were subsequent analyses and visualizations performed on the training set or on the testing set? For the sake of generalizability, the testing set should be considered. And to clarify, was the number of extracted data points not = 16 trials x 200 sequences x 3 time points (= stimulus + delay 1 + delay 2)?

Yes, the analyses were done on the training set; the model performance generalize very well to the testing set with > 99.5% accuracy, and revealed the same representational dynamics. This has been further clarified in the revision (page 19). The data extracted were 16 units x 200 sequences (3200 in total) because we only focused on the second delay timestep for dPCA analyses. This is because we initially reasoned that representation in the late delay would be more stable and more reflective of the prioritization status given that the comparison happens on the subsequent timestep.

- The idea of 'overlapping stimulus events' is not very clear. It could be me but I have difficulties in understanding the following: '(e.g., the n-plus-n + 1 trial and the n + 2-plus-n + 3 trial were plotted but not the n + 1-plus-n + 2 trial; consequently, a total of 1600 trials were plotted).'

This has been changed; please see our response to the similar point raised by Reviewer 2.

Reviewer #4: In their paper, Wan, Menendez, and Postle present rotational remapping as a plausible account for how unprioritized working memory items (UMIs) are neurally represented. Prior work has shown that UMIs may be represented in activity-silent traces and/or as inverted or suppressed representations. This paper takes the approach of using RNNs to try to gain insight into the mechanisms underlying UMI-to-PMI representational transformations. RNNs were trained to perform a 2-back WM task similar to the EEG task from Wan et al. (2020). The authors first use PCA to visualize the hidden layer of the RNNs, showing that the UMI (the currently task-irrelevant item in WM that will become task-relevant on the next trial) is represented similarly but rotated compared to the PMI (the currently relevant, prioritized WM item). Moreover, the representation seems to gradually rotate over time in transforming from the UMI to PMI. They then use a demixed PCA analysis to quantify a rotational index. Similar analyses were performed on the EEG data from Wan et al. (2020), again showing UMI representations rotated relative to PMI. The authors conclude that rotational remapping is a candidate computation for dynamic WM prioritization, and shows promise for artificial neural networks to aid mechanistic interpretations for human cognition.

Overall, the demonstration of rotationally remapped UMIs in both the RNN and EEG data is very interesting! The topic of how WM items of different priorities are represented neurally is important and timely, and I read this paper with great interest. The authors did well in discussing how their findings are bridged to other work, and in raising important theoretical questions in the discussion. That said, I found several aspects of the methods and interpretation confusing and/or lacking in detail, and was left with an overall sense of not really knowing quite what these results

mean in terms of neural representations. If the authors are able to clarify and provide a clearer link between the RNN and EEG data, I think the ideas in this paper would make a nice contribution to the literature.

(Also, note that I am not an expert in RNN methods, so I did not evaluate the details of that aspect of the manuscript.)

Main concerns:

1) What exactly is meant by "rotational remapping"? Is it the idea that the UMI is a rotated version of the PMI? Or is it referring to the dynamic mechanism by which the UMI transforms into the PMI via rotation over time? This is important because the RNN shows both, but the EEG data are only showing the first. Which is the main contribution of the paper?

We believe that our paper's main contribution is to demonstrate that a hypothesized mechanism that we had been calling "priority-based remapping," (and are now calling "priority-based transformation") may indeed be is a general algorithm that brains and machines use to solve the computational problem of needing to hold information in WM but to do so in a way that won't interfere with current behavior that is being guided by different information. Prior to this work we had inferred this from the behavior of multivariate analyses of fMRI and EEG data, but here we demonstrate not only that *it happens but also* how *it happens at the level of the representational code. This is illustrated in the transition of the stimulus-coding axis of the PCA from a decision-potent orientation (when the item in question is a probe) to a decision-null orientation (when it is a UMI) to a decision-potent orientation (when it is a PMI that is needed to guide behavior). Although submitting the data to dPCA allows us characterize these representational transformations quantitatively, it also highlights that our 2-back data only allows us to assess the UMI-to-PMI transition in detail. This is for two reasons. The first is that the variable ISIs makes it impossible to observe* n-n + 1-n + 2 *sequence without a discontinuity between* n-n + 1 *or between* n + 1-n + 2. *More generally, the 2-back task does not include an extended period when an item has PMI status before transitioning to UMI status. (For this, we will need to turn to a retrocuing task.)*

2) I could clearly see the rotational remapping in the RNN representations, but I kept finding myself getting stuck trying to translate that intuitively into what that means in terms of how the brain represents the stimuli.

a. Is the idea that the brain is actually performing computational steps similar to dPCA? If so, what would the RNN hidden layer units reflect in the brain?

We do not think that the brain is "performing dPCA." Indeed, dPCA is not a computation per se, but a dimensionality reduction method that allows us examine the structure of a high-dimensional representation (be it in a brain or in an RNN). By performing the dPCA at multiple timesteps, we get snapshots of the representational geometry at different points in time. If these change from one to another, they indicate that a transformation of this representational geometry has occurred. Determining the transformational matrix between each timestep allows us to map the trajectory of the transformation between the two presumed "end-state" representational formats (for us, a UMI subspace and a PMI subspace) This trajectory is the

priority-based transformation. Having to "talk through this" helped us rethink how to organize the narrative for the revision, and, for example, we now include a summary of this explanation in the Results section.

Additionally, our ability to convey an intuition for this has been aided considerably by R1's suggestion that we draw on the highly relevant study of Panichello and Buschman (2021). In one of their tasks, monkeys first viewed two stimuli, one appearing in the upper visual field and one in the lower, then, with the two in WM, waited to be cued as to whether they'd need to recall the color of the "top" stimulus or the "bottom" one. Data from hundreds of PFC neurons were treated as one high-dimensional value, and PCA indicated that prior to the onset of the retrocue, PFC represented the color of the "top" and the "bottom" item in two separate subspaces (one for each location), and in which color information between them was anti-correlated, presumably to facilitate keeping each color memory distinct. After onset of the retrocue, the cued color transitioned into a third subspace, a "template" subspace in which color representation was highly aligned across the two locations. The authors interpret this sequence as initially holding stimulus information two item-specific subspaces that preserved the details of encoding, then transitioning it to an abstracted representation optimized for guiding the recall response. It may be that the PMI subspace that we have characterized in our study shares a functional role similar to that of the this "template" subspace. A compressed version of this summary and comparison now appears in the Discussion.

b. I also had difficulty trying to intuitively track a single stimulus through this transformation and how it would result in the match/no-match outcome. For example, at time n, a "blue" stimulus is represented in the right side of this representational space. Then it rotates such that that stimulus is now located towards the top of the space during the UMI period 1:2. Then it continues rotating so that it is in the upper left during PMI period 2:2. And then at time n+2 it's rotated back to the "decision"-aligned axis. But it's now in the opposite direction (left side) of the representational space. How would this representation of the stimulus n at time n+2 be compared to the incoming n+2 stimulus to determine a match, and why are the matches clustered in the center of that representational space? Some sort of cartoon walk-through or intuitive explanation would be useful here.

This blow-by-blow is correct, but with one subtle point meriting clarification. From Delay 2:2 to n+2 *it doesn't "rotate back" to where it was at timestep n, rather, at* n+2 *it is approaching the final point of its 180° trajectory through the trial. To refer back again to the comparison to the Panichello & Buschman (2021) paper, this suggests there may be 3 functionally specific subspaces: a "probe" subspace (which doesn't apply to the P&B task), a "item-specific/storage" subspace (for our task, the UMI subspace), and the "template" subspace (for our task, the PMI subspace). Because the PCA doesn't know about different task variables, it also captures decision-related variance in the data. The PCA at timestep* n *captures the coexistence of the "probe" state along the stimulus dimension and the "respond" state along the decision dimension whereas the PCA at time step 2 captures the coexistence of the "PMI" state along the stimulus dimension and the "respond" state along the decision dimension. (Note that on any given trial the "respond state" must be "match" or "nonmatch," which can't be deduced for any one trial when they are labeled by stimulus identity, as they are in the column of Fig. 4 that we are considering here.) Stated another way, if one were to re-label the trials at timestep n + 2 by the identity of stimulus n + 2, the configuration would be identical to the configuration for stimulus n at timestep n, which means that n + 2 and n are in opposite locations of the space. We suspect this outcome is conducive to keeping straight which item is the probe and which is the*

PMI, just as in the P&B data the item-specific subspaces kept the upper and lower items distinct. Finally, depending on whether the decision is "match" or "nonmatch", the representation of n *would either "slide" along the stimulus coding axis to the green center strip in the decision structure, which corresponds to an output of [1], or complete its rotation into the flanking manifold (colored blue), and which correspond to an output of [0] (referring now to Figure 4, Decision column). We don't have better insight into why the matches cluster in the center of space other than that it's a linear representation of the system's nonlinear solution in separating match and nonmatch trials. We have expanded the explanation now in the manuscript.*

c. Is the rotational remapping related to the continuous/circular nature of the orientation space?

It is contained in dynamics of the continuous rotation of the PCA, but can't be extracted from it, due to the PCA's "conflation" of task dimensions. (I.e., the precise location of a dot at any timestep depends not only on its priority status, but also on its status along the decision dimension, and along whatever dimension(s) it is that is(are) captured by the band-like manifolds.) See also our response to R#2's point 6.c.

c. (continued) I know that the RNN was not fed orientations per se, just 6 labeled features, but what information did the RNN have about the relationship between those features? Is it similar to orientation in that feature 2 is more similar to feature 3 than feature 4? (If not, why did it produce a representation showing a seemingly continuous structure across the stimulus types?) Is it circular space like orientation? On an intuitive level, I feel like a rotational remapping of orientation space kind of makes sense, and would produce the inverted IEMs. But if the UMI and PMI were flowers and cows, what would this rotational remapping look like?

The input of the RNN is discrete in nature (one stimulus type is no closer to or farther from any other). This means "rotational remapping" (in our parlance, priority-based transformation) would be the same if UMI and PMI were flowers and cows (discrete) and that, therefore, it is not idiosyncratic to circularly related stimuli. The seemingly continuous structure across stimulus types in RNN hidden layer activity patterns reflects the model's solution to the 2-back task, into which we don't have better insight. Importantly, however, it is arbitrary, in that one for one RNN [0 1 0 0 0 0] and [0 0 0 1 0 0] might be adjacent, but for the next one they won't be.

3) The EEG data seem to only partially confirm the RNN data. A main feature of the RNN results was the timecourse analysis and gradual rotating of the data. Could a timecourse analysis be done on the EEG data? At minimum the delays could be split into 2 timepoints each to mimic the RNN analysis (which might actually clean up the EEG data some if there was continuous rotation). But the EEG data could lend themselves to something even cooler: a timepoint by timepoint analysis with higher temporal resolution, plotting the rotation angle over time.

This is, indeed, an intriguing idea, but due to some of the limitations of the 2-back task that we've discussed elsewhere, we think it could be more profitably applied to dataset using a retrocuing procedure.

4) Also, what does it mean that the EEG data showed rotation with dPCA but not PCA?

As we have discussed in response to an earlier point by this, and other, reviewer(s), and that we (consequently) now emphasize to a greater extent in the revised manuscript, PCA confuses many stimulus-irrelevant sources of variability, which can potentially obscure dynamics that are

restricted to stimulus-relevant subspaces. The many more sources of variability/noise in the EEG data likely swamped the ability of the PCA to reveal this stimulus-specific effect. dPCA, in contrast, is more specific – it finds dimensions that maximize variability between stimulus conditions and thus is able to capture dynamics that are specific to stimulus-specific subspaces.

5) The rotation index (RI) measure:

a. More clarity is needed in describing this measure. I did not follow if lower RI values mean more rotation or a better-fitting rotational structure. For example, if the representation rotates 180° but this rotation is very noisy, would that have a higher or lower RI compared to a representation that rotates 10° but the rotation is extremely precise?

Higher RI would means a "better-fitting rotational structure" but not a greater degree of rotation, which was quantified by the rotational angle metric. The two metrics are independent from each other. So in the reviewer's example, the 10° rotation will have a higher RI than the 180° rotation, albeit a smaller rotational angle. However, these points are moot for this revision, because it no longer includes the RI measure.

b. The authors used permutation testing to determine significance, but it appears the shuffling of labels was done after the dPCA procedure. I'm not sure this is valid. Wouldn't it be better to shuffle the data labels before the dPCA to obtain a true null distribution?

We followed the reviewer's suggestion and obtained results from a permutation method in which we shuffle the labels before the dPCA procedure. The results did not change.

c. On p.17 it says a $p<.05$ would indicate a "pure rotation". Is that a valid statement? Or is it simply indicating a rotation greater than expected by chance?

By "pure rotation" we had meant the extent of rotation that remains after accounting for the non-rotational factors involved in this linear transformation. A "significant rotation" would be captured by a greater-than-chance RI value. (However, these points are also moot for this revision.)

Minor points

6) A simple figure or schematic to visually show the reader the architecture of the RNN would be much appreciated. Also some explanation of how certain parameters were decided (e.g. why 10 RNNs, 7 units, and different numbers of trials & blocks vs human EEG data?)

We have now added a diagram of the RNN architecture (Figure 3). We chose to only train 10 networks as they revealed very similar network behavior and representational dynamics. Networks with other numbers of hidden units (up to 256) gave qualitatively similar results; we chose to use 7 units because they are few enough to solve the task, and the network solutions (as evaluated by representational dynamics) were the most consistent across training instances). These facts have now been added to the manuscript. Number of trials and blocks were selected for pragmatic reasons, and we have no reason to suspect they would influence the results.

7) As someone not an expert in RNNs, it struck me the near-perfect accuracy of the RNN at performing this task. Is that typical? If the human participants are performing the task at a considerably lower accuracy, what does that mean for the comparison? Related point: if only

highly accurate RNNs were included in the analysis, why not perform the EEG analysis with only correct trials included?

Given the power of the LSTM algorithm, it is not surprising for the networks to excel at this simple task that follows an unvarying and perfectly predictable sequence of events. RNN and the human brain are very different dynamic systems, and we do not have a principled way to match or otherwise relate behavioral accuracy between the two model systems. We chose to use all EEG trials because (1) the IEM reconstruction from the EEG study, which inspired this work, used all trials for analysis (2) using all trials instead of just the correct ones affords more statistical power.

8) Figure 2 I had a bit of trouble following the details. What is the scale/units on the color bar? Why is this different from the scale of the y-axis on the reconstructions in the right plots? Also, is channel 0 centered on stimulus "n" at all timepoints? (If so, why isn't there a strong reconstruction of the stimulus during the initial presentation of "n"?)

Based on the comments of all the reviewers, we have completely reworked this figure, and so these questions are no longer relevant.

9) In Figure 3, the black dashed line illustrates the "stimulus coding axis" and the blue dotted line depicts the "decision-based structure". These are very important terms as they are used for interpreting rotational representations (e.g., the decision-based structure becomes perpendicular to the stimulus coding axis) but these terms do not seem to be explicitly defined in the text. How were these axes determined? Computationally, or by eyeballing a best fit on the plots? (Also, what's the significance of the band-like representational structure?)

We now elaborate on these formulations in the text. For the stimulus-coding axis, the Results now include: "(That is, a stimulus's identity can be read out based on its location along this axis. A schematic illustration of this axis is superimposed on some of the timesteps from Figure 4, "stimulus" column, with a black dotted line.)" The same is also done for the decision-coding axis (the terminology that we have now adopted to be consistent with "stimulus-coding axis"). And they were, indeed, determined by "eyeballing," which is the best that we can do with the PCA results. We don't have any insight into the band-like representational structure, although we do now describe it explicitly in the results, and note that at different times during the trial they variously serve as an "organizational substrate" for stimulus identity and for the match/nonmatch decision. With regard to the organization of the decision, all we can do is observe that it's a linear representation of the system's nonlinear solution in separating match and nonmatch trials.

10) For Figure 4B (the EEG data), it's harder to see the patterns. A few suggestions:

a. Consider using color coded dots. I understand grayscale was used so as not to confuse the orientations with the arbitrary RNN conditions, but then it would be more intuitive to color code 4B and have 4A grayscale. In any case, the gray legend is hard to see and compare, it doesn't capture the circular nature, and the connected lines didn't seem to help.

We have followed this suggestion and used color-coded dots for Figure 6, which shows EEG data.

b. Perhaps it would also help to have a third column to visually display the rotated UMI (based on the rot.angle), so readers can directly compare PMI and "rotated UMI"?

This figure relates to the rotation analysis and therefore has been removed from the revision.

c. It would also be helpful to see the plot of the dPCA subspaces next to the means, as in Figure S4.

This figure relates to the rotation analysis and therefore has been removed from the revision.

11) The definitions of stimulus and global variance were not clear.

Stimulus variance captures the portion of data variability only across different stimuli, whereas global variance refers to the overall variability in the data. Please refer to the equations in the text for the exact definition.

12) The linked OSF page is currently empty (but I appreciate the authors making their data available for open-access!).

The materials described in the Data Availability section has been added to the OSF page.
