# Supplementary material for: Priority-based transformations of stimulus representation in visual working memory
Source: PLoS Comput Biol. 2022 Jun 2;18(6):e1009062. doi: 10.1371/journal.pcbi.1009062 (PMC9197029; doi:10.1371/journal.pcbi.1009062)
Supplement: Attachment — Submitted filename: WanMenendezPostle_2022_3rdRevision_responseToReviewers.pdf [file pcbi.1009062.s003.pdf]

Responses to review comments

In this file we have pasted-in the reviews of PCOMPBIOL-D-21-00800 in this Times New Roman font, and interleaved our replies *in this italicized sans serif font*.

* * *

All of the reviewers feel the revised manuscript is significantly improved. However, as detailed in their comments, a few minor concerns remain. In particular, Reviewers 1, 2 and 4 all highlight the importance of clarifying the relationship between the EEG data and the RNNs. Reviewers 1 and 2 suggest clarifying how the nature of EEG recordings may affect the interpretation of the results. Finally, Reviewer 4 notes a difference in the exact task performed by the RNNs and subjects and whether aligning these more closely may change the results.

We appreciate the quick and constructive reviews and address them below. Before getting into the point-by-point responses, we would first like to address a question that underlies all of these reviews, as captured here by R #4: "… the RNN results are neat and interesting, but they really don't match at all with the EEG results… I'm struggling with what it means if an RNN shows something but the neural data do not, and what we can/should take away from that."

We have modified the Abstract and portions of the Introduction (and Discussion) to make clear that the primary goal of this work is not to establish that "RNNs solve the 2-back task in just the same way that humans do (or vice versa)" [our paraphrase]. Rather, it is now specified in the newly added second and third sentences of the Introduction: "… the cognitive system must solve a fundamental computational problem of how to maintain information in a readily accessible state while also preventing it from interfering with ongoing behavior. The primary goal of work presented here is to investigate how this might be accomplished." Additional modifications to the Introduction emphasize that although the possibility of a priority-based representational transformation has been suggested by the results from previous fMRI and EEG studies, this cannot be definitively "proven" by these findings because the MVPA and IEM methods used to analyze those data don't provide direct access to representational codes (more detail on this below, in a response to R#1). Thus, our primary goal was accomplished with the RNN simulations, as now foreshadowed near the end of the Introduction: "This trajectory [visualized with PCA] provided novel, independent evidence that the transition of the functional role of an item from memory probe to UMI to PMI is accompanied by transformations of its representational format." Once this initial goal was achieved, an important, but secondary goal that related to the EEG data, was twofold: 1) to demonstrate that the methods used to interrogate the RNNs can also be applied to neuroimaging data; and 2) to carry out a preliminary assessment of whether the human brain might carry out priority-based transformations with dynamics that resemble those of the RNNs.

Reviewer's Responses to Questions
Comments to the Authors:
Please note here if the review is uploaded as an attachment.
Reviewer #1: While I think the paper has improved after the revision, several of my concerns were not properly addressed, as I detail below (not sorted by relevance).

1. To be clear, I did not suggest the authors reframe the paper.

With respect, the reviewer had named this section of their review "Framing," then proceeded to list concerns under this heading.

My point was that the previous version of the manuscript touched explicitly on the current debate of active vs silent WM right from the outset, but then did not position their results within this debate. In the current manuscript, the authors say 'the present report does not relate to this question', but this is not true. This report aims at clarifying the mechanisms of UMI storage, precisely the aforementioned debate.
That being said, I think the manuscript in the current form is clearer in this regard. I appreciate that the authors now acknowledge in the introduction that they are offering an alternative explanation to the empirical finding of inverted encoding, but I think the sentence 'the present report does not relate to this question' should be removed.

We've now rewritten this section (and in the process removed the offending clause) as follows: "If two items are currently held in WM, one possible solution could be to encode the "unprioritized memory item" (UMI) into a pattern of synaptic weights (Barak & Tsodyks, 2014; Stokes, 2015), an "activity-silent" trace (Myers et al., 2017) that might be less likely to interfere with the currently active "prioritized memory item" (PMI). Although some previous neuroimaging studies have reported that the prioritization of one item held in WM leads to a decrease-to-baseline of the activity level of the UMI (LaRocque et al., 2013; Lewis-Peacock et al., 2012; Rose et al., 2016), whether an "activity-silent" mechanism may contribute functionally to WM remains a topic of vigorous debate (Christophel et al., 2018; Schneegans & Bays, 2017; Sprague et al., 2016; Stokes et al., 2020). In the present report, we evaluate an algorithmically different solution for prioritization: the priority-based transformation of the UMI into a representational format that, although active, is different from that of the PMI. Such a transformation could minimize the likelihood that the UMI interferes with ongoing behavior."

2. My remark about having ignored the apparent contradiction between the findings of Rose et al. and Wan et al. remains an issue. Note that this (and other concerns) should be addressed directly in the manuscript, not in the rebuttal.

Point taken. We've now added the following to the Discussion:
"Our success applying dPCA to an extant EEG dataset in this study suggests that this approach may also provide novel insights if applied to the data from studies that have previously been interpreted as evidence for activity-silent storage mechanisms (e.g., LaRocque et al., 2013; Lewis-Peacock et al., 2012; Rose et al., 2016). The fact that dPCA does not make assumptions about the representational structure of stimuli means that it's possible that it could find evidence for stimulus representation where a model-based approach, such as IEM, has failed. (Indeed, this is what happened with the PMI from the EEG data set in this study – compare Fig. 2 with Fig. 6.)"

3. My remark about the inverted encoding potentially originating from decoding from different areas/sensors was not addressed.

Incorrect, this remark was addressed in the previous round of review. The reviewer had written "I guess inverted decoding in EEG signals would mean that AMI are stored in some area(s) while the UMI are stored in other(s)?" *and we had responded: "This is a possibility that we can't rule in or out with scalp-level EEG. However, we did (and continue to) note in the Discussion that in the Libby and Buschman (2021) study the "sensory axis" and the "memory axis" were represented by the same population of neurons in auditory cortex. Panichello and Buschman (2021) also explicitly call attention to this aspect of their results from PFC. Finally, we also note that, in*

studies with fMRI, priority-based remapping occurred in the same posterior regions that also represent the PMI (van Loon et al, 2018; Yu et al., 2020)"

This is a hypothesis that can be tested directly in the data at hand, without the need to collect more data or train new RNNs.

It is not clear to us that this hypothesis could be tested in a satisfactory way, because of the inverse problem associated with trying to demix and then localize signals on the scalp to sources in the brain. We do plan to assess this hypothesis with fMRI data, which allow for unambiguous region-by-region analyses and comparisons.

4. Why include networks with 7 units after all? The author's justification (default pytorch configuration) is unsatisfactory. Removing these networks from the manuscript would substantially improve the readability of the manuscript.

First, we apologize for creating the impression that our reason for selecting 7 units was that we simply adopted a default PyTorch configuration, this was an inelegant attempt to say that the architecture of the LSTM units that we used was the defaults provided in PyTorch. We have expanded this parenthetical statement into a complete sentence for clarity: "Specifically, we used the default LSTM architecture in PyTorch with its default initializations." We also cleaned up the statement that "We initially chose to use 7 units because this was the smallest number that solved the task with network solutions that were highly consistent across training instances (as evaluated by representational dynamics from the PCA visualization)."

With regard to removing them from the manuscript, we prefer not to because we believe that their comparison versus the (newly added) results from 60 hidden-unit RNNs trained on a circular stimulus set are of considerable interest. This is because the 7-hidden-unit networks are trained on a set of unrelated one-hot vector stimuli, yet direct comparison shows that the representational dynamics of the two networks are nearly identical, despite the differences in dimensionality (7 vs. 60) and stimulus set (unrelated vs. metrically related and circular). This speaks to the generality of the priority-based transformation – it may be a fundamental operation that is invariant across these different network parameters.

5. The main claim of this report is the link between data and the RNN. I appreciate the transparency in the abstract about this link (or the lack thereof), but I still think figure 5 and 6 (perhaps the main results?) are rather obscure and don't transmit the idea that data and RNN are comparable - quite the opposite?

As clarified in the response to the Editor that preceded these point-by-point responses, "the primary goal of this work is not to establish that "RNNs solve the 2-back task in just the same way that humans do (or vice versa)." Thus, we hope that comparison between Figures 5 and 6 is now less obscure, and accomplishes both "secondary goals" that we also articulated above: demonstrating that the methods used to interrogate the RNNs can also be applied to neuroimaging data; and carrying out a preliminary assessment of the extent to which the human brain might carry out priority-based transformations with dynamics that resemble those of the RNNs.

5. In the new version of the manuscript, I was a surprised by the sentence 'Thus, although MVPA and IEM produced the results that gave rise to the priority-based remapping hypothesis, [they] are poorly suited to evaluating it, because they don't permit direct measures of neural representation', because the limitation is not in the decoding method but the nature of the EEG data.

This sentence no longer appears in the re-revised manuscript. However, in the interest of a fully transparent exchange of ideas, we have to say that we disagree with the reviewer's argument here. MVPA is a method for classification of patterns in the data. It is true that with MVPA results one can draw inferences about representational structure, e.g., one might assume different codes for categories A vs. B if a decoder trained on A cannot generalize to B. But this is not the same as directly observing the representational structure. IEM assumes a priori knowledge of the representational bases of the stimuli being studied. When it works, one can assume that the a priori model is a good approximation of at least one aspect of the true representational format, but there are limitations to how far the results can be interpreted. Indeed, although we have previously applied our preferred interpretation to findings of flipped IEM reconstructions (Yu, Teng, & Postle, 2020; Wan et al. 2020) we have never been able to rule out alternative possibilities, including the possibility that they were artifacts of the IEM procedure that didn't tell us anything about representational transformation.

Reviewer #2: Wan et al have substantially revised their paper. The rotational mappinng no longer features in the EEG data, and instead a scale re-mapping is used. The story is different but remains highly topical. Many parts of the text have been clarified.

My only suggestion is to make the abstract more explicit about the main finding. I think their interpretation is that RNNs effectively perform smooth rotation, preserving information, while EEG signals could suggest a scaling down / collapsing.

We have modified the abstract accordingly.

This would help highlight the importance of their nice results. Just to interpret further, though not wanting to be too controversial, I wonder if the new analysis suggests that information that is "out of mind" is "out of sight", at least from EEG.

Yes, this is indeed intriguing, but framing it this way brings us uncomfortably close to the question that is a primary concern of Reviewer #1, which is whether these results speak to the idea of the activity-silent representation of information ("out of sight"). Instead, we think that the spirit of this suggestion is met by a new insight that we are including for the first time in this second revision: dPCA may offer a novel and powerful way to assess when an active trace of the PMI has vanished from WM.

Reviewer #3: Thank you for your responses to my review. The authors have answered all my questions and responded quite well to my comments. I find this revision along with the very interesting additions (eg scaling, dispersion, UMI-PMI-decision angles) satisfactory.

Reviewer #4: This manuscript is a revised version of one I previously reviewed (Reviewer 4). The authors have clearly put a lot of work into this revision, which is impressive and commendable. The manuscript now includes much clearer descriptions of the methods and results, and the conclusions seem more in line with the data. So in that sense, the authors have addressed most of my specific concerns and I find the manuscript much improved.

However, the resulting changes to the manuscript now paint a picture where the RNN results are neat and interesting, but they really don't match at all with the EEG results. Again, this is handled well in the revision: the results are organized into clear comparisons/hypotheses, and the discussion doesn't over-extend the findings. But now I'm struggling with what it means if an RNN shows something but the neural data do not, and what we can/should take away from that.

As noted at the beginning of this file, this statement captures what we think is the single biggest outstanding disconnect between ourselves and the reviewers, and we hope that the response that we provided to this question satisfactorily addresses it.

A few specific issues I find myself getting hung up on:
- The manuscript now clarifies that the RNN was trained on 6 discrete features with arbitrary relationships, and this differs from the continuous circular relationship between the 6 orientations of the EEG experiment. But, why? This seems a fundamental difference between the two. I get that the authors wanted to have the RNN results not be idiosyncratic to the circularly related stimuli, but that only makes sense if they found similar results across the RNN and EEG. Given that the results are now quite different, I'm a bit lost in how much stock to put in the RNN findings. It feels like there should be a comparison model where the 6 features of the RNN are coded circularly and continuously, to see if these representational transformations persist in that context.

We are grateful to the reviewer for this observation because, not unlike the observations of R#2 during the first round of review (that prompted us to explore scalar transformation as an alternative to rotation), repeating these simulations but with a set of circularly dependent stimuli allowed for a broader interpretation of our results. That is, repeating the 60-D RNN simulations with a set of circularly dependent stimuli revealed that, although the dependencies within the stimulus set are captured by the RNN, the dynamics of the priority-related transformation do not change. This is an important benchmark for future research on the control of the contents of WM.

- I may have missed this in the authors response, but why do the EEG data in the revised manuscript (Fig 6) look so different from the initial manuscript (Fig 4)? In the original manuscript, the EEG projections seemed to transform/rotate substantially between the UMI and PMI representations, but now that doesn't really seem to happen. As described in the revision, there's no suggestion of a reversal and the angle separating them is much smaller. Can one even conclude from the EEG data that there's a transformation / progression in representational state from UMI to PMI? I feel like I'm missing something.

In our initial submission (i.e., PCOMPBIOL-D-21-00800) our analyses treated the two delay periods (i.e., item n *plus its subsequent delay and item* n + 1 *plus its subsequent delay) as two discrete phases of the trial, and so we epoched and baselined the data accordingly. For the revised manuscript, inspection of the results of our initial applications of scalar transformation analyses called our attention to the fact that it's problematic to track a transformational trajectory across a discontinuity, and so we "started again from scratch," re-epoching the data into continuous time series of "delayA-stimulus-delayB". Doing this required us to baseline the data differently, which explains the difference in stimulus representations between the original manuscript and the revisions. (More specifically, we now realize in retrospect that the procedure of using the delay immediately preceding each stimulus as the baseline for each item-*n*-plus-its-subsequent-delay inadvertently biased the IEM reconstruction of each PMI, because the baseline for these data contained the UMI!)*

So overall I'm not sure what to think about this manuscript. The authors have put a great deal of work into an improved revision, and I really do want to acknowledge that, despite my reservations.
