# Supplementary material for: Priority-based transformations of stimulus representation in visual working memory
Source: PLoS Comput Biol. 2022 Jun 2;18(6):e1009062. doi: 10.1371/journal.pcbi.1009062 (PMC9197029; doi:10.1371/journal.pcbi.1009062)
Supplement: Attachment — Submitted filename: WanMenendezPostle_2022_4thRevision_ResponseToReviewers.pdf [file pcbi.1009062.s004.pdf]

Responses to editors's request for further revisions

In this file we have pasted-in the operative text from your Decision on your article submitted to PLOS Computational Biology (PCOMPBIOL-D-21-00800R2), on 3 May 2022, in this Times New Roman font, and interleaved our replies *in this italicized sans serif font*.

* * *

Therefore, we hope you would be willing to further revise the Introduction and Discussion of the manuscript to place greater emphasis on a) the general motivation that led you to compare computational models and the brain

In the Introduction, we now spell out with more granularity our motivation for carrying out computational simulations to develop a better understanding of brain function. To summarize, the two extant computational models that explicitly address flipped decoding/model reconstruction in neuroimaging data leave an important explanatory gap, because both are only capable of assessing one class of algorithmic operation, which is to vary the level of attentional activation. In order to assess the possibility that an alternative class of algorithmic operation – representational transformation – may be employed to solve the computational problem of prioritization, we needed to employ a modeling framework (RNNs with LSTM units) that is capable of carrying out such transformations. Furthermore, successfully measuring these transformations in the RNN with dPCA validated this analytic approach for assessing evidence of representational transformation in human EEG data. Here's the newly revised text [manuscript pp. 8-9]:

"It is instructive to consider the two models reviewed above from the perspective of the framework of Marr and Poggio (1976): They address distinct computational problems – prioritization within WM (Manohar et al., 2019) vs. removal from WM (Lorenc et al., 2020) – they propose different algorithmic solutions – inhibition via biased competition (Manohar et al., 2019) vs. excitation paired with selective gain modulation (Lorenc et al., 2020) – yet they observe similar patterns of neural implementation – flipping. Of particular relevance for our interests here is that although the details of their algorithmic operations differ, both models are constrained to finding only one class of solution: changing the strength of attention. Importantly, neither allows for the alternative that we will test here, which is the transformation of an item's representational geometry.

Previous WM research has implicated representational transformation as a solution to a third computational problem for WM: the retention of information in the face of distraction (e.g., Libby & Buschman, 2021; Parthasarathy et al., 2017). Our goal with the present work was to explore the possibility that the computational problem of prioritization in WM might also be solved algorithmically via representational transformation… By comparing the stimulus representational schemes embedded in the EEG and RNN data, we hope to reveal whether humans and RNNs might employ similar algorithmic principles. Given that the RNNs are optimized to solve the 2-back task, we can also potentially use the RNN results to evaluate whether the algorithm that humans use reaches optimality."

and b) discussing and interpreting the differences between the model and neural data.

To accomplish this, we have reorganized the Discussion such that, although the propositional content is more-or-less the same, it is now reorganized with a general overview, then a

subsection entitled “Comparison of RNN vs. EEG results” that is further divided into sub-subsections entitled “Similarities” and “Differences” and finally a subsection entitled “Contributions and limitations of the current work.” These new revisions begin on manuscript page 34:

*“**Comparison of RNN vs. EEG results***

Similarities

- *Stimulus representation in both RNN and EEG data went through a priority-based transformation, occupying, in turn, two distinct subspaces (UMI and PMI). This indicates that the UMI was actively represented in both RNN and EEG (c.f., LaRocque et al., 2013; Lewis-Peacock et al., 2012; Rose et al., 2016). Importantly, it confirms that, at the algorithmic level, prioritization in WM is carried out, at least in part, by an operation of representational transformation.*
- *The representational trajectories of RNN and EEG data are indicative of an active transformation, and so cannot be accounted for by inhibition (c.f., Manohar et al., 2019).*
- *The angles between UMI and PMI subspaces (RNN: 84°, EEG: 77°), and between UMI and decision subspaces (RNN: 81°, EEG: 82°) were similar for RNN and EEG. These patterns are consistent with a process that might minimize the influence of the UMI on other concurrent operations, including the retention of the PMI and the processing of the probe.*

Differences

- *Unlike the RNN data, the EEG data did not show evidence of a sign reversal of the best-fitting scalar transform. It remains to be determined if this reflects a fundamental difference in how the human brain carries out priority-based remapping, or if it may reflect a limitation of extracranial EEG. (E.g., the dynamics of priority-based transformations are different in different brain areas of the NHP (Panichello & Buschman, 2021), but comparable inter-regional differences would be mixed in our whole-scalp EEG data.)*
- *In the EEG data, the group-average stimulus representation transformed into its final configuration the PMI subspace earlier than in the RNN data (Figures 5 and 6). (Indeed, human subjects, on average, recoded item n into its UMI and PMI configurations simultaneously, and then later prepared for item n + 2 by collapsing the UMI structure during delay 2, whereas the RNNs recoded the item around the time when the priority status of the stimulus changed.) It is also noteworthy that the rate of this transformation was highly variable across individual EEG datasets, but not across RNNs. An important question for future research is whether individual differences in this factor may relate to behavioral performance, as well as whether it is sensitive to such factors as strategy or reward contingency.*
- *For the EEG data, the angle between PMI and decision subspaces was 83°, whereas for the RNN it was 35°. This pattern in the RNN data is consistent with close alignment of these two subspaces that might facilitate comparison of the PMI and the probe. Similar to the point raised previously, future work is needed to determine whether this difference reflects an important difference in decision-making between human and RNN, or if it is a consequence of poor spatial resolution of the EEG data. (E.g., the effects of selection on WM information are markedly stronger in the PFC than in the visual cortex of NHPs (Panichello & Buschman, 2021).)”*
